# Fabrication of flexible UV nanoimprint mold with fluorinated polymer-coated PET film

**DOI:** 10.1186/1556-276X-6-458

**Published:** 2011-07-18

**Authors:** Ju-Hyeon Shin, Seong-Hwan Lee, Kyeong-Jae Byeon, Kang-Soo Han, Heon Lee, Kentaro Tsunozaki

**Affiliations:** 1 Department of Materials Science and Engineering, Korea University, Anam-dong 5-ga, Seongbuk-gu, Seoul 136-713, South Korea; 2 Asahi Glass Co., Ltd., Research Center, 1150 Hazawa-cho, Kanagawa-ku, Yokohama-shi, Kanagawa 221-8755, Japan

## Abstract

UV curing nanoimprint lithography is one of the most promising techniques for the fabrication of micro- to nano-sized patterns on various substrates with high throughput and a low production cost. The UV nanoimprint process requires a transparent template with micro- to nano-sized surface protrusions, having a low surface energy and good flexibility. Therefore, the development of low-cost, transparent, and flexible templates is essential. In this study, a flexible polyethylene terephthalate (PET) film coated with a fluorinated polymer material was used as an imprinting mold. Micro- and nano-sized surface protrusion patterns were formed on the fluorinated polymer layer by the hot embossing process from a Si master template. Then, the replicated pattern of the fluorinated polymer, coated on the flexible PET film, was used as a template for the UV nanoimprint process without any anti-stiction coating process. In this way, the micro- to nano-sized patterns of the original master Si template were replicated on various substrates, including a flat Si substrate and curved acryl substrate, with high fidelity using UV nanoimprint lithography.

## Introduction

In order to form micro- to nano-sized patterns, various lithographic technologies have been used, such as DUV photolithography [1], e-beam lithography [2,3], X-ray lithography [4,5], laser holographic lithography [6], nanosphere lithography [7], scanning probe microscopy lithography [8], and so on. Except for DUV photolithography, these conventional lithography technologies require either a complicated patterning system with a high process cost or offer limited throughput and, thus, are not suitable for mass production. None of these technologies allow micro- to nano-sized patterns to be formed on a non-flat surface. Recently, nanoimprint lithography [9-11] has emerged as one of the most effective technologies to fabricate micro- to nano-sized patterns. Due to its low process cost and high throughput, nanoimprint technology can be used for the mass production of nano-sized patterns [12,13].

UV nanoimprint templates need to have high stiffness in order for the nano-sized protrusion patterns to be transferred to the substrate and sufficient flexibility for conformal contact to be achieved over a large-sized substrate. Flexible templates can be applied to non-planar substrates. In addition, high transparency to UV is required for the template to be used for UV nanoimprint lithography. A sufficiently low surface energy is also necessary to avoid the need for an anti-sticking coating on the template, which would require the extra deposition of a Si oxide layer [14,15],

In this study, a fluorinated polymer layer was coated on a flexible polyethylene terephthalate (PET) film, since micro- to nano-sized patterns can easily be formed on a fluorinated polymer layer by the hot embossing process [16,17], and fluorinated polymers have a very low surface energy [18,19]. With this fluorinated polymer-coated flexible PET mold, micro- to nano-sized patterns were fabricated on a flat Si substrate and curved acryl substrate with high fidelity using UV nanoimprint lithography.

## Experimental procedure

### Fabrication of flexible UV nanoimprint mold

**Figure 1:**
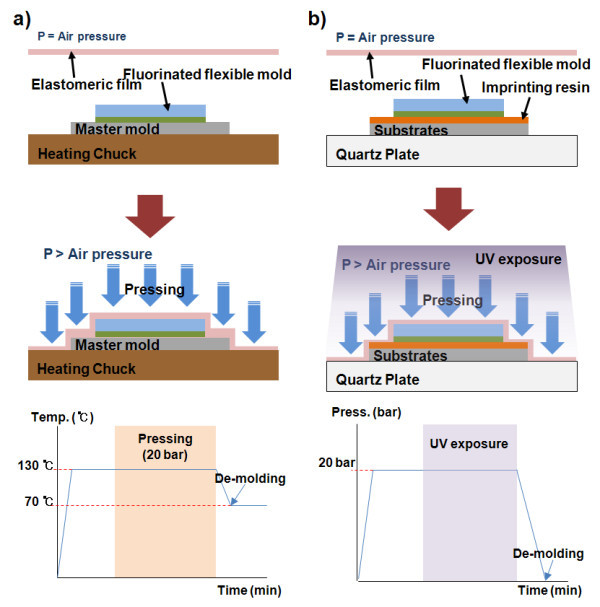
**Schematic drawing**. Schematic drawing of (a) hot embossing lithography system and (b) UV nanoimprint lithography system.

Figure 1 shows experimental schematics and detailed process flow of hot embossing lithography system and UV nanoimprint lithography system made by NND (Seoul) in Korea. Both systems used to fabricate nano-sized patterns are of the vessel type.

**Figure 2:**
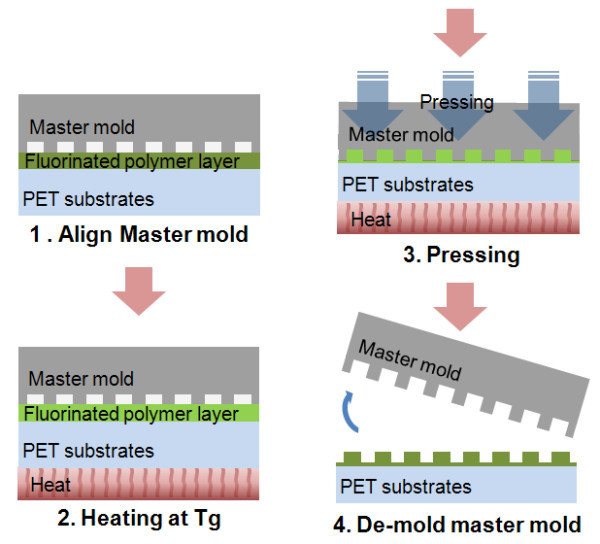
**Fabrication of UV nanoimprint template**. Fabrication using hot embossing process of PET film coated with fluorinated polymer layer.

Figure 2 shows the overall fabrication process of the UV nanoimprint template using the hot embossing process of a PET film coated with a fluorinated polymer layer. An aligned stack consisting of the master Si mold and PET film was loaded in the UV nanoimprint system, as described elsewhere [20] and heated up to 130nu°C. A pressure of 20 bars was applied to fill the cavity of the Si master mold with the fluorinated polymer. After cooling to 70°C, the Si master mold was demolded from the patterned fluorinated polymer-coated flexible PET mold. Finally, reversed patterns were formed on the fluorinated polymer-coated flexible PET film.

**Figure 3:**
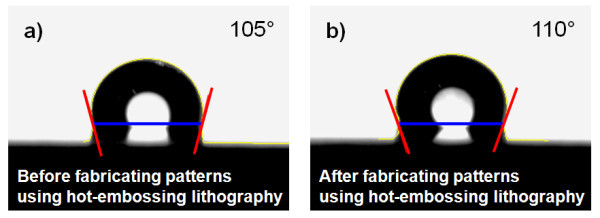
**Change of contact angle by fabricated pattern using hot embossing lithography**. (a) Before fabricating patterns and (b) after fabricating patterns.

The contact angles of the unpatterned and hot-embossed fluorinated polymer surfaces are shown in Figure 3. Prior to the hot embossing process used to form the nano-sized patterns, the contact angle of the unpatterned fluorinated polymer surface was 105°. After the hot embossing process, the contact angle of the fluorinated polymer-coated flexible PET mold was increased to 110°. This result demonstrates that the surface energy of the fluorinated polymer was inherently high, so that it can be used as an imprinting mold to fabricate micro- to nano-sized patterns without the need to coat it with an anti-stiction layer.

### Imprinting process using replicated flexible UV nanoimprint mold

**Figure 4:**
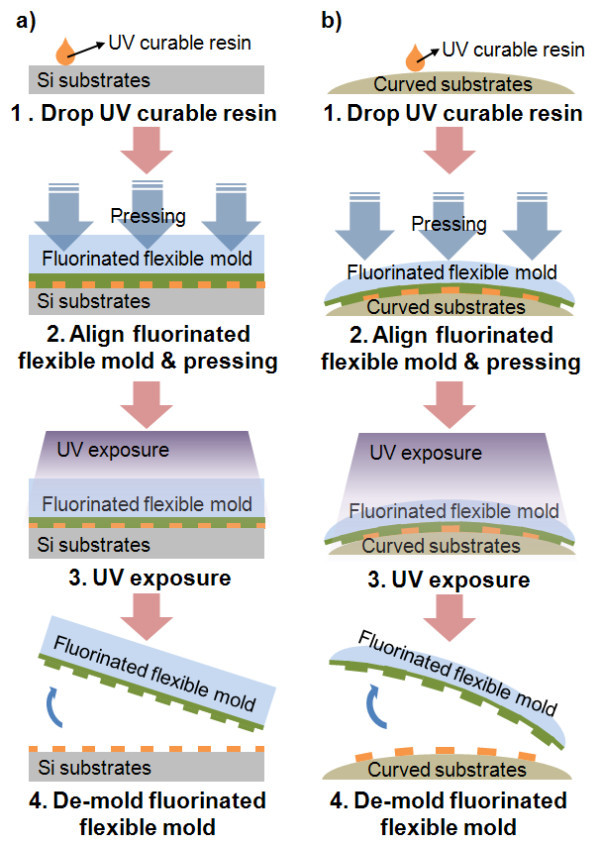
**Imprinting process using replicated fluorinated polymer-coated flexible PET mold**. (a) Imprinted on flat Si substrates and (b) imprinted on curved acryl substrates.

A hot-embossed flexible PET mold was used as a template for UV nanoimprint lithography without the coating of an anti-stiction layer. As shown in Figure 4a,b, the UV nanoimprint process was performed on both a flat Si wafer and curved acryl substrate. The same imprinting system as that employed for the hot embossing process of the fluorinated polymer-coated flexible PET mold was used. A monomer-based UV curable resin, NIP-K28™, made by the ChemOptics Company (Daejeon, South Korea) was used. As shown in a previous report [21], an isotropic pressure was applied through a flexible membrane to assure uniform pressing between the PET film mold and substrate. Due to the flexibility of the PET mold, conformal contact can be achieved between the PET mold and curved substrate, and a uniform pressing force can be delivered. A pressure of 20 bars and UV light with a wavelength of 365 nm were used in the imprinting process.

## Results and discussion

### Photographic images

**Figure 5:**
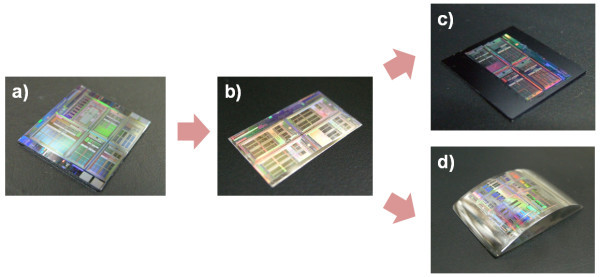
**Photographic images**. (a) Si master mold, (b) hot-embossed fluorinated polymer-coated flexible PET film, (c) imprinted resist patterns on flat Si substrates using hot-embossed PET film shown in b, and (d) imprinted resist patterns on curved acryl substrates using hot-embossed PET film shown in (b).

Figure 5a,b,c,d shows the photographic images of the Si master mold, hot-embossed fluorinated polymer-coated flexible PET film and imprinted resist patterns on the flat Si substrate and curved acryl substrate made using the hot-embossed PET film, respectively. Both the hot embossing and UV nanoimprint patterning processes were done on large size substrates without any noticeable defects.

### SEM micrographs

**Figure 6:**
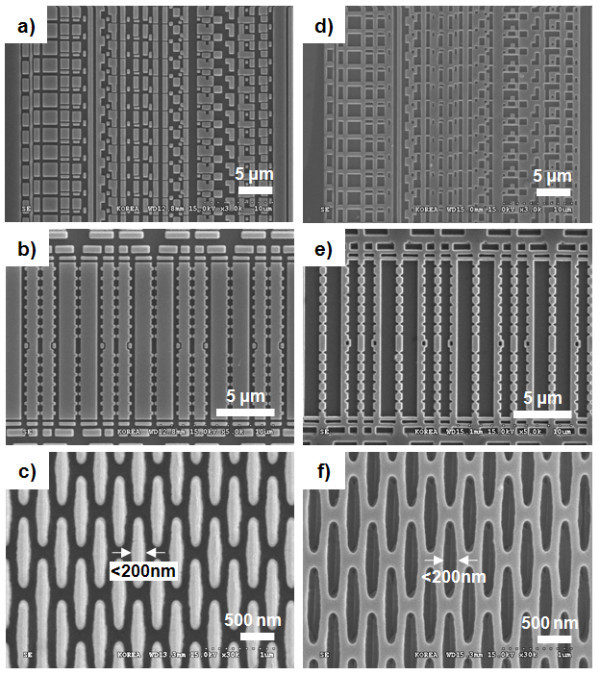
**SEM micrographs**. (a, b, c) micro- and nano-sized patterns on master Si mold, (d, e, f) replicated micro- and nano-sized patterns on fluorinated polymer-coated flexible PET film by hot embossing lithography.

Figure 6 shows the scanning electron microscopy (SEM) micrographs of the micro- and nano-sized patterns on the master Si mold and replicated patterns on the fluorinated polymer-coated flexible PET film by hot embossing lithography. An S-4300 SEM system from Hitachi was used. As shown in Figure 6, the micro- and nano-sized patterns on the master Si mold were replicated onto the fluorinated polymer-coated flexible PET film by hot embossing lithography with high fidelity and without any defects. Due to the slightly tapered profile of the patterns of the Si master mold and elastic nature of the hot embossing process of the fluorinated polymer, the hot-embossed patterns on the PET films were slightly smaller than the patterns of the Si master mold.

**Figure 7:**
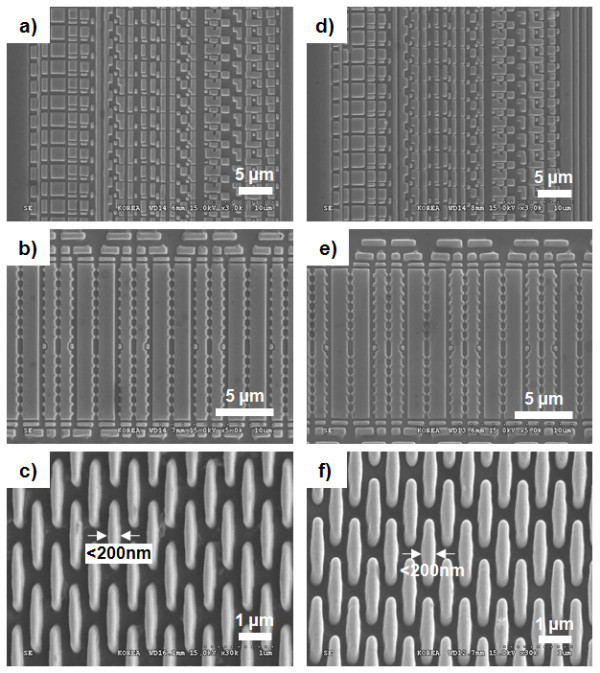
**SEM micrographs of imprinted resist patterns by UV nanoimprint lithography**. Using hot-embossed fluorinated polymer-coated PET film, (a, b, c) imprinted resist patterns on a flat Si substrate and (d, e, f) imprinted resist patterns on a curved acryl substrate.

**Figure 8:**
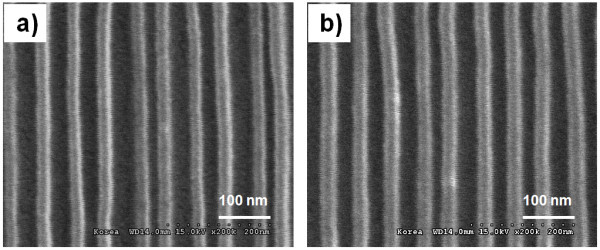
**SEM micrographs of imprinted 20- to approximately 30-nm-sized patterns by UV nanoimprint lithography**. Using hot-embossed fluorinated polymer-coated PET film (a) imprinted patterns on a flat Si substrate and (b) imprinted patterns on a curved acryl substrate.

The SEM micrographs of the imprinted micro- and nano-sized patterns made by UV nanoimprint lithography using the hot-embossed fluorinated polymer-coated PET film are shown in Figure 7. The hot-embossed flexible PET film, coated with the fluorinated polymer, was used as the UV imprint mold. Figure 7a,b,c show the imprinted resist patterns on the flat Si substrate using UV nanoimprint lithography. The shape and size of the micro-sized, complex patterns of the Si master mold were replicated with high fidelity on the flat Si substrate. Even sub-200-nm-sized nanopatterns were able to be finely replicated. Figure 7d,e,f shows the imprinted resist patterns on the curved acryl substrate. Due to the uniform pressing of the flexible PET film mold over the curved substrate, micro- and nano-sized patterns were able to be successfully imprinted on a curved acryl substrate. These results imply that a hot-embossed flexible PET film, coated with a fluorinated polymer layer, can be used as a mold for the UV nanoimprint lithography of various substrates, including non-planar ones. Furthermore, 20- to approximately 30-nm-sized line/space patterns were fabricated on the flat Si substrate and on the curved acryl substrate. As shown in Figure 8, these patterns were fabricated very finely.

## Conclusions

The micro- and nano-sized surface protrusion patterns of the master template were transferred with high fidelity to the flexible PET film, coated with the fluorinated polymer material, by the hot embossing process.

Since the surface energy of fluorinated polymers is as high as 105° for DI water, a flexible PET film with a patterned fluorinated polymer can be used as a stamp for the UV nanoimprint process without the need for an anti-stiction coating.

Due to the uniform pressing of the flexible PET film mold over either the flat Si wafer or curved acryl substrate, the micro- and nano-sized patterns of the embossed PET film were successfully imprinted onto the substrates using the UV nanoimprint process.

## Competing interests

The authors declare that they have no competing interests.

## Authors' contributions

JHS carried out overall experiments including nanoimprint lithography works as the first author.

SHL was in charge of hot embossing experiment using Si master mold.

KJB carried out the fabrication of Si mold.

KSH was in charge of self-assembled monolayer coating of Si mold

HL conducted design and analysis of all experiments as a corresponding author.

KT made fluoro-resin coated PET film which was used as a substrate for hot embossing process.
